# Confidence maps: statistical inference of cryo-EM maps

**DOI:** 10.1107/S2059798320002995

**Published:** 2020-03-25

**Authors:** Maximilian Beckers, Colin M. Palmer, Carsten Sachse

**Affiliations:** a Structural and Computational Biology Unit, European Molecular Biology Laboratory (EMBL), Meyerhofstrasse 1, 69117 Heidelberg, Germany; b Scientific Computing Department, Science and Technology Facilities Council, Research Complex at Harwell, Didcot OX11 0FA, United Kingdom; c Ernst-Ruska Centre for Microscopy and Spectroscopy with Electrons 3/Structural Biology, Forschungszentrum Jülich, 52425 Jülich, Germany; d JuStruct: Jülich Center for Structural Biology, Forschungszentrum Jülich, 52425 Jülich, Germany

**Keywords:** electron cryo-microscopy, signal detection, false-discovery rate, family-wise error rate, cryo-EM maps, local resolution, *CCP-EM*, software

## Abstract

Confidence maps provide complementary information for interpreting cryo-EM densities as they indicate statistical significance with respect to background noise. They can be thresholded by specifying the expected false-discovery rate (FDR), and the displayed volume shows the parts of the map that have the corresponding level of significance. Here, the basic statistical concepts of confidence maps are reviewed and practical guidance is provided for their interpretation and usage inside the *CCP-EM* suite. Limitations of the approach are discussed and extensions towards other error criteria such as the family-wise error rate are presented. The observed map features can be rendered at a common isosurface threshold, which is particularly beneficial for the interpretation of weak and noisy densities. In the current article, a practical guide is provided to the recommended usage of confidence maps.

## Abstract 2

The concept of statistical signal detection by controlling the false-discovery rate (FDR) to aid the atomic model interpretation of cryo-EM density maps is reviewed. The recommended usage of the FDR software tool is presented together with its successful integration into the *CCP-EM* suite.

## 1. Introduction   

The 3D structure obtained from an electron cryo-microscopy (cryo-EM) experiment corresponds to the Coulomb potential in 3D space of the macromolecule of interest in three dimensions (Frank, 2006 ▸). Electron micrographs of ice-embedded macromolecules are generated by the interaction of elastically scattered and unscattered electrons with the biological specimens in the cryo-microscope (Glaeser, 2016 ▸). At the core of the structure-determination process is the 3D image-reconstruction procedure, which requires the computational determination of the orientations of thousands of individual particle images with respect to a 3D model. Owing to noise from solvent scattering, optical aberrations, imperfect detectors and other sources, inaccuracies arise in the alignment process and the reconstructed maps contain errors in addition to the electrostatic potential. Moreover, inherent molecular flexibility and heterogeneity such as the incomplete stoichiometry of protein complexes contribute to incoherent averages and systematic variation in map values. Compensation for the resulting decay of amplitudes at high resolution is therefore required. For the interpretation of high-resolution map features, a *B*-factor sharpening approach is applied and combined with a signal-to-noise based weighting of the amplitudes (Rosenthal & Henderson, 2003 ▸). While this approach enhances the relevant map signal, it also bears the danger of enhancing noise by oversharpening. Local map variation exacerbates this problem as the optimal sharpening *B* factor varies across the map. Therefore, recent sharpening approaches take into account local amplitude information from a refined atomic model (Jakobi *et al.*, 2017 ▸).

The interpretation of cryo-EM maps is most challenging initially when atomic reference structures are missing. Regardless of the applied sharpening or filtering routine, the precise map values have to be treated with caution in order to avoid the interpretation of noise artefacts as true density variation. Commonly, maps are visualized by thresholding to create 3D isosurface renderings. These are overlaid with atomic models or used to build polypeptide chains by using a series of interactive map tools (Goddard *et al.*, 2018 ▸; Emsley & Cowtan, 2004 ▸). Ideally, the threshold is chosen such that signal is displayed and noise is removed, which is generally expressed as a multiple of the standard deviation σ beyond the map noise. In contrast to crystallographic maps, however, this σ value varies strongly for different cryo-EM maps owing to the dependence on the ratio of particle to volume size, which prevents σ values from being used universally. Moreover, thresholding the cryo-EM map reduces the information content to a binary detection of the voxel and discards more accurate representations of the electrostatic potential of the macromolecule. Once the atomic model has been initially built, atomic coordinate refinement requires the complete dynamic range of the determined cryo-EM densities in order to accurately model different atomic masses, positions and conformations.

To develop a more robust framework for associating the values of a cryo-EM map with significance, we have recently presented a statistical framework based on multiple hypothesis testing and false-discovery rate (FDR) control, which transforms the cryo-EM map into a new volume that we term a confidence map (Beckers *et al.*, 2019 ▸). Similar approaches are routinely used in other imaging domains, for example fMRI imaging (Genovese *et al.*, 2002 ▸; Lohmann *et al.*, 2018 ▸). Confidence maps contain detection errors with respect to background noise and can be thresholded by controlling the FDR in the detected signal. Confidence maps provide complementary cryo-EM map information that is particularly helpful for the interpretation of weak and ambiguous signal close to background-noise levels. In this *CCP-EM* Spring Symposium article, we review the basic principles of confidence maps and focus our presentation on practical aspects and extensions of the procedure as it is now integrated in the *CCP-EM* software suite.

## 2. Testing for significant signal with respect to background noise   

We refer to noise in cryo-EM maps as any incorrect modification of the signal from the true electrostatic potential of the structure of interest. Multiple sources of noise that accumulate in a cryo-EM experiment have been discussed previously (Penczek *et al.*, 2006 ▸). For a concise treatment of cryo-EM noise in the context of confidence maps, we refer readers to Beckers *et al.* (2019 ▸). Although noise levels in the solvent region can be assumed to be higher than the noise in the particle region owing to solvent displacement, we show that we can use solvent map values outside the particle to estimate the noise for the following statistical analysis (Beckers *et al.*, 2019 ▸). For each voxel in the 3D map, we conduct a statistical hypothesis test for positive deviations from background noise. Given an estimate of the cumulative distribution function of the background distribution, which is obtained by assuming a Gaussian distribution or using a nonparametric procedure, *p*-values are calculated for each voxel as the probability under the null hypothesis of having an intensity at the respective voxel at least as great as the observed background.

## 3. False-discovery rate control of cryo-EM maps   

Conducting a statistical test for each voxel within the map results in a multiple testing problem. A consequence that arises from the multiplicity is that many tests can give rise to false-positive detections, and this problem is more severe for large numbers of tested hypotheses. A widely used framework to deal with the large-scale multiple testing problem is false-discovery rate (FDR) control (Benjamini & Hochberg, 1995 ▸). The approach adjusts the statistical significance level to set an upper bound on the expected proportion of false discoveries. Mathematically, the FDR is defined as
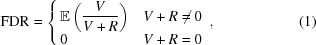
where *V* is the number of false positives and *R* is the number of true positives. In the statistical literature it is usually stated that the FDR is ‘controlled at level α’ if the true FDR is smaller than α. Typical FDR-controlling approaches take the *p*-values of the individual tests and transform them to satisfy the FDR criterion. These FDR-adjusted *p*-values can then be thresholded at a specified FDR level. For confidence maps, we further invert the adjusted *p*-values for visualization purposes, *i.e.* thresholding of a confidence map at 0.99 means a maximum FDR of 1%. As an FDR-controlling procedure, we use by default the method of Benjamini and Yekutieli, which has the advantage of controlling the FDR under arbitrary dependencies between the *p*-values (Benjamini & Yekutieli, 2001 ▸). Cryo-EM maps possess artificial correlations as a result of the 3D reconstruction and post-processing, which statistically can be considered arbitrary dependencies.

## 4. Generation of confidence maps   

**Figure 1:**
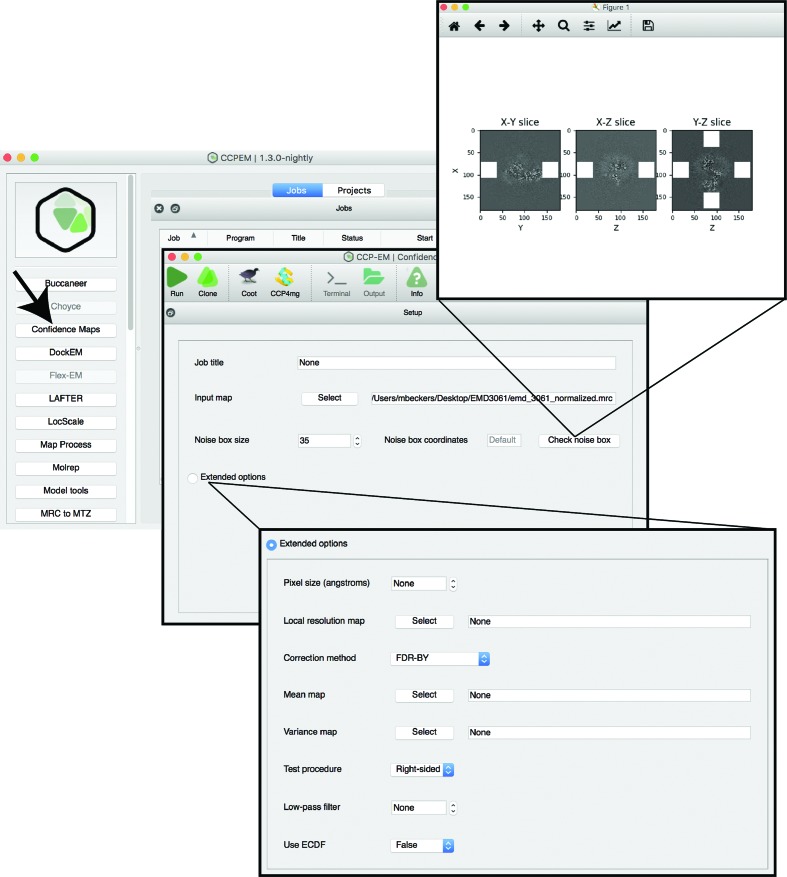
The graphical user interface (GUI) for confidence-map generation in *CCP-EM*. The main GUI, visualization of noise slices and extended options are shown. A simple ‘Check noise box’ button allows direct visualization of the noise cubes.

The only required input for computing a confidence map is an unmasked and globally sharpened cryo-EM map. The approach is applied to sharpened EM maps, as unsharpened maps lack high-resolution features as well as noise. Therefore, using unsharpened maps will result in underestimated background noise estimation. Typically, the required input is generated by common post-processing procedures in the respective image-processing programs (Rosenthal & Henderson, 2003 ▸; Scheres, 2012 ▸; Punjani *et al.*, 2017 ▸; Desfosses *et al.*, 2014 ▸). Within this map, the default background estimation uses a total of four map cubes from the solvent area outside the particle (Fig. 1 ▸). In principle, the size of the map cubes should be maximized to increase the sample size for reliable background noise estimation. At the same time, one should avoid including particle density into the cubes as this will bias noise estimation. Identifying regions outside the structure is straightforward for single-particle maps, whereas cryo-EM densities obtained by subtomogram averaging often do not have a clearly solvent-isolated structure. Therefore, particular care needs to be taken to specify the location of the cube for noise estimation in such nonstandard cases. We implemented the option to specify a manual cube location as well as the volume size (Fig. 1 ▸). The *CCP-EM* GUI allows interactive adjustment of the noise cubes; clicking the ‘Check noise box’ button opens an image with three slices through the map with noise areas labelled in white. In this way, the cube parameters can be adjusted to select suitable background regions. Moreover, the slice views can be used to identify cases when noise levels are not uniform over the map and when, therefore, confidence-map generation should be avoided.

## 5. Local resolution measurements can be included for the generation of confidence maps   

The statistical power of the FDR thresholding approach can be increased by the incorporation of local resolution information, which is available as an extended option in the *CCP-EM* GUI. The procedure of specifying the noise cube location and the size of the cubes is identical to that described above. In addition to the cryo-EM map, the user provides a map containing the local resolution values at the respective voxel positions, which is the standard output of a series of programs for local resolution estimation (Heymann & Belnap, 2007 ▸; Scheres, 2012 ▸; Hohn *et al.*, 2007 ▸; Kucukelbir *et al.*, 2014 ▸). Using local resolution estimates, the cryo-EM map can be locally low-pass filtered, which improves the appearance of the map features as dominant noise in local regions is removed (Cardone *et al.*, 2013 ▸). Consequently, the signal-to-noise ratios are also increased locally. Tracking the positional background-noise levels after local filtering enables the generation of confidence maps using local resolution information. Very low-resolution artefacts can arise, leading to smeared densities in the confidence maps extending over the whole box. To avoid this, it can be beneficial to restrict the resolution range of the local resolution map to reasonable values, for example from 2 to 20 Å for a 3 Å map. The incorporation of local resolution information is particularly useful in the presence of substantial local resolution variations, as global sharpening and filtering usually lead to the undersharpening of lower resolution features and the oversharpening of higher resolution features. The resulting confidence maps capture such areas side by side, including the highest resolved parts of the structure, and guide the user through the density-interpretation steps within a single confidence map. Owing to the increase of statistical power, we find that more stringent error levels can be applied for thresholding confidence maps generated using local resolution information.

## 6. Case studies: Tobacco mosaic virus, a bacterial ATP synthase and a eukaryotic ribosome   

**Figure 2:**
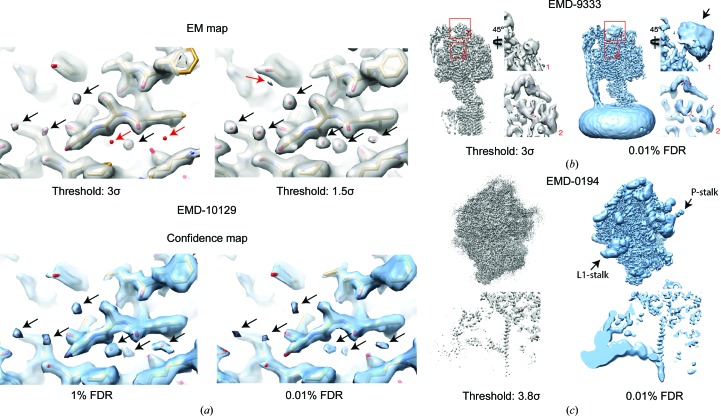
Confidence maps facilitate the interpretation of cryo-EM maps. (*a*) Comparison of cryo-EM and confidence maps at different thresholds for a 1.9 Å resolution TMV reconstruction (EMD-10129). While the cryo-EM map has a strong threshold dependence (top), leading to different map peaks for water molecules (red arrows), the confidence map allows reproducible assignment of water molecules over different significance levels (bottom). (*b*) Locally filtered cryo-EM map (EMD-9333) of a bacterial ATP synthase (left) with the corresponding confidence map (right), including two magnified views corresponding to the red boxes in the molecule overview. The arrow points to density that is likely to correspond- to a 10×His tag. (*c*) Cryo-EM map of a eukaryotic ribosome (EMD-0194, left) together with the confidence map (right), including two magnified views.

In a recent article (Weis *et al.*, 2019 ▸), confidence maps were used to assign the structural details of the disassembly switch of Tobacco mosaic virus (TMV). Several decades earlier, Don Caspar had proposed a switch mechanism of conformational changes driven by carboxylate interactions (Caspar, 1964 ▸), but the precise residue location was still missing owing to the flexibility of the respective residues and the absence of two comparative structures in the ON/OFF switch states. Structure determination of two data sets acquired under conditions mimicking the extracellular and intracellular conditions resulted in two maps at 2.0 and 1.9 Å resolution in water and at high Ca^2+^/acidic pH, respectively. The confidence maps allowed the assignment of significant cryo-EM density for the respective residues and showed that multiple conformations of the involved residues are supported by the map recorded in the water condition. Further analysis and validation by means of an additional Ca^2+^/acidic pH cryo-EM map revealed that the switch exists in two distinct structural states. Moreover, using the confidence maps the authors were able to place 71 and 91 water molecules per monomer. In these cases, we placed the water molecules based on the detected confidence map peaks, the expected molecular size and the proximity to the protein structure. Even in high-resolution regular cryo-EM maps this is still a daunting task, as noise peaks can be easily mistaken for waters without further validation (Fig. 2 ▸ *a*, top). The confidence maps, however, enabled placement by means of statistical significance (Fig. 2 ▸ *a*, bottom).

To further illustrate the utility of confidence maps including local resolution information, we generated two maps for examples from the EMDB with local resolution variation from near-atomic up to nanometre. Comparison of the map of a locally filtered bacterial ATP synthase map (EMD-9333; Guo *et al.*, 2019 ▸) with the corresponding confidence map shows improved overall interpretability using the statistical thresholding approach (Fig. 2 ▸ *b*). For the locally filtered map, low-resolution parts such as the stalk domain remain missing at low σ thresholds, while at the same threshold high-resolution parts have already become noisy. The confidence map enables the interpretation of the complete complex at a low FDR of 0.01%, showing the appearance of significant low-resolution density corresponding to a 10×His tag (Fig. 2 ▸ *b*, bottom right). In another example, given by a eukaryotic ribosome (EMD-0194; Juszkiewicz *et al.*, 2018 ▸), the expansion segments and the ribosomal stalks display lower resolution, as is typical for eukaryotic ribosome structures (Fig. 2 ▸ *c*). In the deposited map, these parts are oversharpened and appear discontinuous owing to noise, which is a result of the global sharpening and filtering. Generating the confidence map by including local resolution information shows the respective domains clearly, including both high- and low-resolution features visible at a single threshold of 0.01% FDR.

## 7. Visualization of confidence maps   

**Figure 3:**
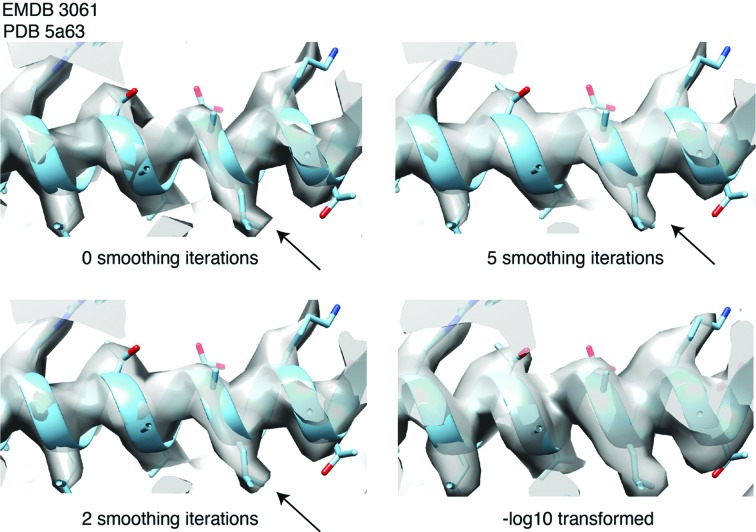
Visualization of confidence maps with additional smoothing. Confidence maps have an almost binary contrast, which can lead to sharp edges when rendered as an isosurface (top), as shown for the map EMD-3061. The appearance can be improved in most visualization programs. For example, in *UCSF Chimera*, additional surface smoothing can be used to make the appearance less sharp and more cryo-EM map-like (middle, bottom). Additionally, a −log_10_ transformation of the FDR values can be used to smoothen the gradients for visualization.

Confidence maps can be displayed at a given FDR threshold in common visualization programs that use an isosurface rendering approach. A typical property of such a map is that when density can be clearly distinguished from background noise, voxels assume values close to 1, which results in a close-to-binary distribution of signal versus background. Consequently, when visualizing confidence maps, for example in *UCSF Chimera* (Pettersen *et al.*, 2004 ▸), they appear different from common cryo-EM maps. Confidence maps will display very sharp voxel features, with almost all values close to the extremes of 1 and 0. In order to make them appear like typical cryo-EM maps, the displayed surface can be oversampled and smoothed (Fig. 3 ▸). Alternatively, a −log_10_ transformation of the FDR values leads to more shallow gradients and allows detailed analysis at very small FDRs (Fig. 3 ▸, bottom).

## 8. Assessing additional error criteria for confidence maps   

Multiple testing is a major field of research in statistical inference (Wilson, 2019 ▸; Zhang *et al.*, 2019 ▸; Ignatiadis *et al.*, 2016 ▸), and several additional error rates and error-controlling procedures beyond Benjamini–Yekutieli FDR control have been proposed. The family-wise error rate (FWER) specifies the probability of having false positives at all (Lehmann & Romano, 2005 ▸). We have implemented FWER for confidence-map generation as an additional option within the *CCP-EM* suite. In contrast to FDR, FWER is considered to be the strictest criterion to rule out false-positive detection. Procedurally, background-noise estimation and *p*-value calculation remain identical, while only the FDR correction is replaced by a FWER-controlling routine. With respect to FWER-based confidence maps, thresholding at a value of 0.99 then means a probability of 1% of having any false positives at all.

Mathematically, the FWER is defined as
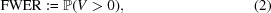
where *V* denotes the number of false-positive hypotheses.

Although controlling the FDR in confidence maps already facilitates the interpretation of voxels in terms of significance, we still expect false positives, *i.e.* up to as many as is specified by the FDR threshold: in the case of 100 000 significant voxels, 1% FDR corresponds to an expected maximum of 1000 false-positive voxels. Controlling the FWER instead of the FDR may be desirable in cases where no false positives can be tolerated for the interpretation. Generic methods for the control of FWER and FDR are given by the Bonferroni–Holm (Holm, 1979 ▸) and the Benjamini–Yekutieli (Benjamini & Yekutieli, 2001 ▸) approaches. It can be shown that both criteria control the respective error rates under arbitrary dependencies between the tested *p*-values (Benjamini & Yekutieli, 2001 ▸; Holm, 1979 ▸).

**Figure 4:**
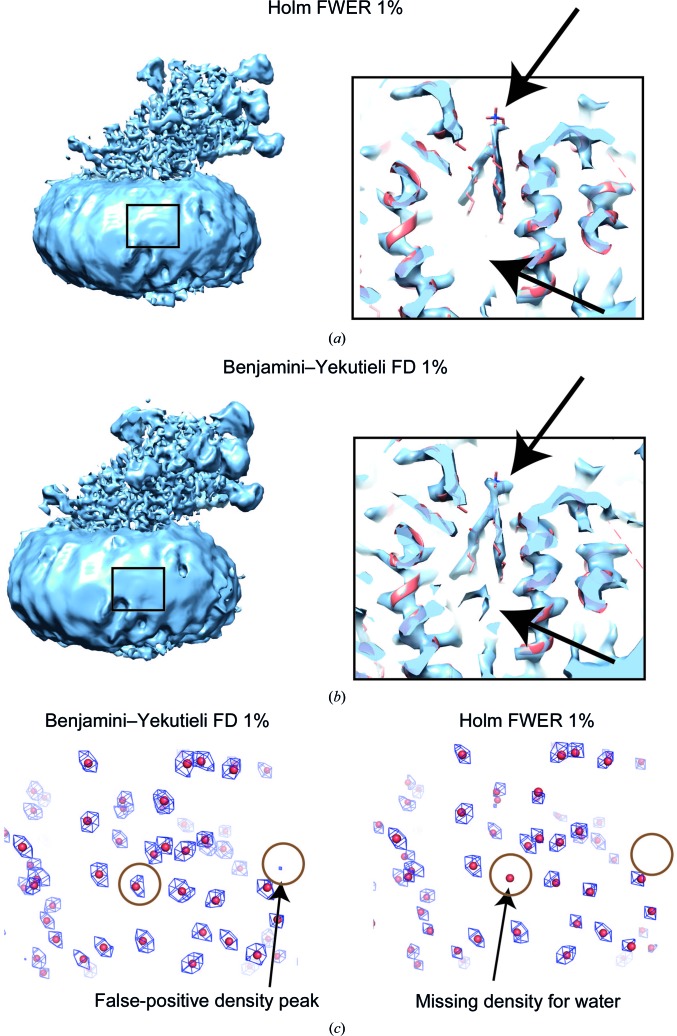
Comparison of FWER- and FDR-controlling procedures in confidence maps. Confidence maps based on (*a*) Holm FWER and (*b*) Benjamini–Yekutieli FDR are compared for a γ-secretase map (EMD-3061). Respective inset enlargements in the interior of the transmembrane domain are shown on the right. (*c*) Comparison of detected false-positive voxels and false-negative water molecules for a simulated map of 4194 water molecules (taken from PDB entry 6cvm).

In order to investigate the different error criteria from the multiple testing approaches in the context of cryo-EM maps, we compared the FDR and FWER using the 3.4 Å resolution cryo-EM map (EMD-3061) of γ-secretase (Bai *et al.*, 2015 ▸). As expected, FWER control is more stringent at 1% as less density is declared significant compared with 1% FDR (Figs. 4 ▸ *a* and 4 ▸ *b*). For example, the presumably false-positive density below the lipid declared by the 1% FDR thresholding is not present after 1% FWER thresholding. In addition, signal is assigned to the head of the embedded lipid (top arrow) using the FDR criterion, whereas this density appears smaller at 1% FWER. In order to assess the performance of different error-rate thresholding criteria more quantitatively, we applied them to a simulated map of 4194 water molecules (taken from PDB entry 6cvm; Bartesaghi *et al.*, 2018 ▸). The simulated map was generated using *UCSF Chimera* and we included Gaussian white noise with a standard deviation of 0.5. The resulting signal-to-noise ratio is 1.75 for the density peaks and corresponds to common noise levels in cryo-EM maps in the high-resolution shells. We compared the detected false-positive map peaks that do not originate from water molecules and also the number of missed water molecules for the different procedures at 1% FDR or 1% FWER, respectively. At 1% FWER we did not detect any false positives, whereas at 1% FDR some false-positive peaks were identified, which could be mistakenly interpreted as water (Table 1 ▸). However, the decreased number of false positives in the case of FWER control comes at the price of missing a small number of water molecules (*i.e.* there are some false negatives). With respect to the analysis of cryo-EM maps, FWER provides the error criterion that is most useful for the interpretation of weak and isolated signal, as occurs for water molecules and bound ligands in high-resolution structures (Table 1 ▸). To compare FDR-based and FWER-based confidence maps, FDR is usually more useful for initial interpretation and FWER for the in-depth analysis of more ambiguous parts. Both error criteria provide complementary information in addition to cryo-EM maps that can be used during the model-building process.

## 9. Discussion   

Confidence maps are based on the statistical framework of multiple hypothesis testing. Applying global error rates in a multiple testing setting such as cryo-EM maps is a complex task as dependencies between the individual voxels have to be considered (for the treatment of dependencies, see Beckers *et al.*, 2019 ▸). Thus, control of the errors should only be carried out rather conservatively, which means that a confidence map at an FDR of 1% will have a true FDR of below 1%. When applying a threshold to confidence maps, the FDR provides an error criterion that is theoretically interpretable: for example, 1% FDR corresponds to 1% of the thresholded density being background noise. Using a series of test cases of deposited cryo-EM maps, we demonstrated that the 1% FDR threshold was sufficient to visualize the relevant molecular details (Beckers *et al.*, 2019 ▸). When incorporating local resolution information, lower FDRs of down to 0.01% can be used successfully (Fig. 2 ▸).

The stringency of the FDR criterion is, in principle, also related to the types of features to be interpreted. For clear continuous density stretches such as polypeptide conformations, the occurrence of single false-positive voxels rarely complicates the interpretation. However, when interpreting point densities such as water and ions the cost of false-positive hits can be substantially higher. In these cases, more restrictive thresholds should usually be chosen. This will be particularly relevant as higher true atomic resolution cryo-EM maps become available. An additional possibility to aid the interpretation of atomic resolution densities may be the FWER criterion introduced here, which further minimizes the expected rate of false positives. Taken together, we conclude that lower FWER or more stringent FDR thresholds should be applied when more confident statements about the observed densities are to be made. Although the threshold of a confidence map can be adjusted by the user, it is much less sensitive to the inclusion of noise compared with threshold adjustments of cryo-EM maps. For common cryo-EM maps, the applied threshold is difficult to interpret in terms of significance. Specific σ thresholds remain highly subjective owing to the multitude of errors in the cryo-EM experiment and the subsequent 3D image-reconstruction procedure, as described in Beckers *et al.* (2019 ▸). Confidence maps, however, suppress noise, and the associated FDR threshold provides a quantitative error criterion with respect to background noise and gives feedback on the validity of the detected density features.

Confidence maps provide complementary information that should be used together with the original density map. As confidence maps contain detection probabilities, information about scattering strength and occupancies is no longer present. Therefore, confidence maps must not be used for atomic coordinate refinement. However, for manual building and initial assignment of the atomic model, confidence maps directly provide information on which density parts can be faithfully analyzed, and incorporation of this additional information into automated model-building protocols may prove to be beneficial in the future.

Confidence maps aim to separate signal from background noise. If significant signal is detected in a voxel, it means that, up to the specified confidence level, it is neither background from the amorphous ice nor shot noise. As such, signal corresponds to every feature that contains contributions beyond background noise. However, the following limitations need to be considered where confidence-map generation including background estimation was procedurally successful but claimed incorrect features as signal. In the case when a large fraction of particles were mis­aligned and included in the reconstruction, they will inevitably give rise to statistical assignment of signal in a generated confidence map with no structural meaning. Similar effects will occur in cases of highly preferred orientations and overfitting of noise. As confidence maps are able to detect very weak signal, in particular in cases where local resolutions are provided, this type of incorrect signal will be more prominent in confidence maps compared with regularly sharpened cryo-EM maps. Although this property of the signal-detection approach bears the danger of overinterpretation, it is also sensitively reveals more general problems of the computed cryo-EM structure. Owing to the significant recent progress in improved image quality and image-processing routines in cryo-EM, these cases are becoming less prominent, but internal image-processing validation criteria still need to be applied to ensure the determination of reliable cryo-EM structures. Background-noise determination is critical and is still a step that requires user intervention when confidence maps are generated. Estimation of noise levels in the solvent area can only provide accurate measurements if the background noise is homogeneous over the whole map. This remains an approximation owing to various factors discussed in our previous work (Beckers *et al.*, 2019 ▸), but it controls the significance level even when the background-noise level is overestimated.

Moreover, the manual selection of noise areas may affect this process and adds a level of subjectivity, especially for the case of subtomogram averages. Although guidelines can be given to the user on how to carefully apply the parameters (see above), in the future we envision the full automation of these parts of confidence-map generation. Once automated routines are available, the process of confidence-map generation can be seamlessly integrated into the initial building of atomic models. The first step towards this goal is the implementation of confidence-map generation in the *CCP-EM* suite (available now in version 1.4). This way, the complementary map information from confidence maps is available at little computational cost and integrated in common map interpretation and atomic modelling tools.

## Figures and Tables

**Table 1:** Comparison of detected false-positive voxels and false-negative H_2_O molecules for a simulated map of 4194 H_2_O molecules (taken from PDB entry 6cvm) The numbers of false-negative H_2_O molecules and false-positive voxels together with the true FDR that this corresponds to are given.

Controlling procedure	False-positive voxels	False-negative H_2_O molecules
Holm FWER 1%	0/FDR: 0.00%	18 of 4194
Benjamini–Yekutieli FDR 1%	7/FDR: 0.05%	0 of 4194

## References

[bb1] Bai, X.-C., Yan, C., Yang, G., Lu, P., Ma, D., Sun, L., Zhou, R., Scheres, S. H. W. & Shi, Y. (2015). *Nature*, **525**, 212–217. 10.1038/nature14892 PMC4568306 26280335

[bb2] Bartesaghi, A., Aguerrebere, C., Falconieri, V., Banerjee, S., Earl, L. A., Zhu, X., Grigorieff, N., Milne, J. L. S., Sapiro, G., Wu, X. & Subramaniam, S. (2018). *Structure*, **26**, 848–856. 10.1016/j.str.2018.04.004 PMC6129192 29754826

[bb3] Beckers, M., Jakobi, A. J. & Sachse, C. (2019). *IUCrJ*, **6**, 18–33. 10.1107/S2052252518014434 PMC6327189 30713700

[bb4] Benjamini, Y. & Hochberg, Y. (1995). *J. R. Stat. Soc. B*, **57**, 289–300.

[bb5] Benjamini, Y. & Yekutieli, D. (2001). *Ann. Stat.* **29**, 1165–1188.

[bb6] Cardone, G., Heymann, J. B. & Steven, A. C. (2013). *J. Struct. Biol.* **184**, 226–236. 10.1016/j.jsb.2013.08.002 PMC3837392 23954653

[bb7] Caspar, D. L. D. (1964). *Adv. Protein Chem.* **18**, 37–121. 10.1016/s0065-3233(08)60268-5 14151998

[bb8] Desfosses, A., Ciuffa, R., Gutsche, I. & Sachse, C. (2014). *J. Struct. Biol.* **185**, 15–26. 10.1016/j.jsb.2013.11.003 24269218

[bb9] Emsley, P. & Cowtan, K. (2004). *Acta Cryst.* D**60**, 2126–2132. 10.1107/S0907444904019158 15572765

[bb10] Frank, J. (2006). *Three-Dimensional Electron Microscopy of Macromolecular Assemblies: Visualization of Biological Molecules in Their Native State.* Oxford University Press.

[bb11] Genovese, C. R., Lazar, N. A. & Nichols, T. (2002). *Neuroimage*, **15**, 870–878. 10.1006/nimg.2001.1037 11906227

[bb12] Glaeser, R. M. (2016). *Methods Enzymol.* **579**, 19–50. 10.1016/bs.mie.2016.04.010 27572722

[bb13] Goddard, T. D., Huang, C. C., Meng, E. C., Pettersen, E. F., Couch, G. S., Morris, J. H. & Ferrin, T. E. (2018). *Protein Sci.* **27**, 14–25. 10.1002/pro.3235 PMC5734306 28710774

[bb14] Guo, H., Suzuki, T. & Rubinstein, J. L. (2019). *eLife*, **8**, e43128. 10.7554/eLife.43128 PMC6377231 30724163

[bb15] Heymann, J. B. & Belnap, D. M. (2007). *J. Struct. Biol.* **157**, 3–18. 10.1016/j.jsb.2006.06.006 17011211

[bb16] Hohn, M., Tang, G., Goodyear, G., Baldwin, P. R., Huang, Z., Penczek, P. A., Yang, C., Glaeser, R. M., Adams, P. D. & Ludtke, S. J. (2007). *J. Struct. Biol.* **157**, 47–55. 10.1016/j.jsb.2006.07.003 16931051

[bb17] Holm, S. (1979). *Scand. J. Stat.* **6**, 65–70.

[bb18] Ignatiadis, N., Klaus, B., Zaugg, J. B. & Huber, W. (2016). *Nat. Methods*, **13**, 577–580. 10.1038/nmeth.3885 PMC4930141 27240256

[bb19] Jakobi, A. J., Wilmanns, M. & Sachse, C. (2017). *eLife*, **6**, e27131. 10.7554/eLife.27131 PMC5679758 29058676

[bb20] Juszkiewicz, S., Chandrasekaran, V., Lin, Z., Kraatz, S., Rama­krishnan, V. & Hegde, R. S. (2018). *Mol. Cell*, **72**, 469–481. 10.1016/j.molcel.2018.08.037 PMC6224477 30293783

[bb21] Kucukelbir, A., Sigworth, F. J. & Tagare, H. D. (2014). *Nat. Methods*, **11**, 63–65. 10.1038/nmeth.2727 PMC3903095 24213166

[bb22] Lehmann, E. & Romano, J. (2005). *Testing Statistical Hypotheses*. New York: Springer.

[bb23] Lohmann, G., Stelzer, J., Lacosse, E., Kumar, V. J., Mueller, K., Kuehn, E., Grodd, W. & Scheffler, K. (2018). *Nat. Commun.* **9**, 4014. 10.1038/s41467-018-06304-z PMC6167367 30275541

[bb24] Penczek, P. A., Yang, C., Frank, J. & Spahn, C. M. T. (2006). *J. Struct. Biol.* **154**, 168–183. 10.1016/j.jsb.2006.01.003 16510296

[bb25] Pettersen, E. F., Goddard, T. D., Huang, C. C., Couch, G. S., Greenblatt, D. M., Meng, E. C. & Ferrin, T. E. (2004). *J. Comput. Chem.* **25**, 1605–1612. 10.1002/jcc.20084 15264254

[bb26] Punjani, A., Rubinstein, J. L., Fleet, D. J. & Brubaker, M. A. (2017). *Nat. Methods*, **14**, 290–296. 10.1038/nmeth.4169 28165473

[bb27] Rosenthal, P. B. & Henderson, R. (2003). *J. Mol. Biol.* **333**, 721–745. 10.1016/j.jmb.2003.07.013 14568533

[bb28] Scheres, S. H. W. (2012). *J. Struct. Biol.* **180**, 519–530. 10.1016/j.jsb.2012.09.006 PMC3690530 23000701

[bb29] Weis, F., Beckers, M., Hocht, I. & Sachse, C. (2019). *EMBO Rep.* **20**, e48451. 10.15252/embr.201948451 PMC6831999 31535454

[bb30] Wilson, D. J. (2019). *Proc. Natl Acad. Sci. USA*, **116**, 1195–1200.

[bb31] Zhang, M. J., Xia, F. & Zou, J. (2019). *Nat. Commun.* **10**, 3433. 10.1038/s41467-019-11247-0 PMC6668431 31366926

